# Probability of Alzheimer’s disease based on common and rare genetic variants

**DOI:** 10.1186/s13195-021-00884-7

**Published:** 2021-08-17

**Authors:** Valentina Escott-Price, Karl Michael Schmidt

**Affiliations:** 1 Dementia Research Institute, Division of Psychological Medicine and Clinical Neurosciences, School of Medicine, Cardiff University, Hadyn Ellis Building, Maindy Rd, Cardiff, CF24 4HQ UK; 2 School of Mathematics, Cardiff University, Senghennydd Road, Cardiff, CF24 4AG UK

**Keywords:** Alzheimer’s disease, Disease risk, Polygenic risk score, Rare variants, APOE

## Abstract

**Background:**

Alzheimer’s disease, among other neurodegenerative disorders, spans decades in individuals’ life and exhibits complex progression, symptoms and pathophysiology. Early diagnosis is essential for disease prevention and therapeutic intervention. Genetics may help identify individuals at high risk. As thousands of genetic variants may contribute to the genetic risk of Alzheimer’s disease, the polygenic risk score (PRS) approach has been shown to be useful for disease risk prediction. The *APOE*-*ε*4 allele is a known common variant associated with high risk to AD, but also associated with earlier onset. Rare variants usually have higher effect sizes than common ones; their impact may not be well captured by the PRS. Instead of standardised PRS, we propose to calculate the disease probability as a measure of disease risk that allows comparison between individuals.

**Methods:**

We estimate AD risk as a probability based on PRS and separately accounting for APOE, AD rare variants and the disease prevalence in age groups. The mathematical framework makes use of genetic variants effect sizes from summary statistics and AD disease prevalence in age groups.

**Results:**

The AD probability varies with respect to age, *APOE* status and presence of rare variants. In age group 65+, the probability of AD grows from 0.03 to 0.18 (without APOE) and 0.07 to 0.7 (APOE e4e4 carriers) as PRS increases. In 85+, these values are 0.08–0.6 and 0.3–0.85. Presence of rare mutations, e.g. in *TREM2*, may increase the probability (in 65+) from 0.02 at the negative tail of the PRS to 0.3.

**Conclusions:**

Our approach accounts for the varying disease prevalence in different genotype and age groups when modelling the *APOE* and rare genetic variants risk in addition to PRS. This approach has potential for use in a clinical setting and can easily be updated for novel rare variants and for other populations or confounding factors when appropriate genome-wide association data become available.

**Supplementary Information:**

The online version contains supplementary material available at 10.1186/s13195-021-00884-7.

## Introduction

Genome-wide association studies (GWAS) identified genetic risk variants of late onset “sporadic” disease beyond the *APOE* locus [1–4], followed by exome chip analyses identifying rare variants with moderate risk [5–7]. While causal fully penetrant mutations almost certainly lead to development of the disease [8], most of the identified singular nucleotide polymorphism (SNP) risk alleles have not been proven to be causal but replicated as carrying an increased disease risk.

The PRSs are designed to aggregate genome-wide genotype data into a single variable indicating genetic liability to a disorder or trait. PRS studies often reach sufficiently high statistical significance to suggest trait polygenicity and, although the prediction accuracy is usually insufficient for clinical utility [9], PRS has been suggested as a useful tool for the selection for clinical trials of individuals of European ancestry across different traits [10–13]. The PRS prediction accuracy of risk for Alzheimer’s disease (AD) is comparatively high, especially when the diagnosis is based upon pathology confirmed rather than clinical assessment (AUC up to 84%) [14].

Designed to capture the risk of common variants, the PRS aggregates the effects of known genome-wide associated loci [15] and of loci that do not reach genome-wide statistical significance. However, the PRS may not well reflect the effect of rare variants in *TREM2*, *PLCG2*, *ABI3*, *SORL1* [5–7] and very rare highly penetrant mutations in *APP*, *PSEN1* and *PSEN2* [16], as the cumulation of many SNPs of small effect sizes tends to mask the strong effect of a single variant. As LD between rare and common variants tends to be small—for example, the maximum *r*^2^ between a rare variant with minor allele frequency (MAF) = 0.01 and a typical common SNP with MAF = 0.2 is *r*^2^ = 0.04 when the rare alleles of both variants appear on the same haplotype (i.e. D’ = 1) [17]—rare variants and PRS are likely to be independent.

Variants in the *APOE* gene highly affect the AD risk (OR = 3.2, MAF = 0.14) [2]. *APOE* is also associated with lower odds of reaching the over 90th percentile age [18] as it modifies the age at onset; for example, the age at onset of AD for *ε*4*ε*4 carriers is ~ 68 years [19]. Also, *ε*4*ε*4 carriers are more likely to develop other conditions associated with lower life expectancy such as cardiovascular disease and diabetes [20]. People with AD diagnosed in their late 60s live on average 7 years after the clinical diagnosis, whereas AD diagnosis after age 90 is associated with an expected survival of only 2.8 years [21, 22]. Since age is the major confounding factor to the AD risk, it is difficult to disentangle the ageing and disease pathogenic components.

There is little research on whether *APOE* and PRS can be modelled as independent variables. Leonenko et al. [23] show that *ε*4 frequency decreases with age in both cases and controls, whereas the PRS values are higher in older AD patients, indicating a negative correlation in cases, but apparently not in controls. These contravariant effects in cases cancel out when a PRS is formed including *APOE* alongside other SNPs, so subsequent adjustment for age is ineffective. The use of *APOE* genotypes and the PRS (calculated without *APOE*) as two separate predictors accounts for this effect and increases the case/control prediction accuracy but cannot be extended to disease prediction in age groups with different disease prevalence. The approach suggested in the present study accounts both for the age related *APOE* effect and different disease prevalence. We propose estimating the disease probability (between 0 and 1) based on the PRS while accounting separately for high effect size variants and rare highly penetrant mutations. We show the utility of our probability calculations in application to AD.

Our calculations only require the mean and variance of PRS in cases and in controls and the disease prevalence as reference data, thus avoiding the need to share background sensitive genetic data. While the PRS from different studies (with different SNP selection and/or standardisation) cannot be directly compared, disease probability, as a general quantity, can be used for comparative prioritisation of individuals.

## Material and methods

### PRS distribution

The PRS aggregates the effects of multiple genetic markers identified by GWAS. Generally, the PRS is expected to be higher in cases than in controls, indicating a higher genetic risk for the disorder, but the difference in mean PRS between case and control samples may be small. It is important to note that the PRS calculated for an individual does not provide an absolute measure of risk and is meaningless except in relation with the distribution of PRS in cases and non-cases in the underlying population.

The polygenic risk score for individual *j* ∈ {1, …, *N*_*ind*_} is ${PRS}_j=\frac{1}{N_{snps}}{\sum}_{i=1}^{N_{snps}}{g}_{ij}{\beta}_i$, where *N*_*ind*_ and *N*_*snps*_ are the numbers of individuals and of SNPs contributing to the PRS, respectively, *g*_*ij*_ ∈ {0, 1, 2} is the genotype of SNP *i* for individual *j*, and *β*_*i*_ is the effect size (logarithm of the odds ratio or logistic regression coefficient) of SNP *i* in an independent GWAS for the disease. The sample mean and variance are

1 $$ m(PRS)=\frac{1}{N_{ind}}{\sum}_{j=1}^{N_{ind}}{PRS}_j\kern1em \mathrm{and}\kern1em \operatorname{var}(PRS)=\frac{1}{N_{ind}}{\sum}_{j=1}^{N_{ind}}{\left({PRS}_j-m(PRS)\right)}^2. $$

### Estimation of PRS distribution parameters for unscreened controls

Our calculations require the distribution parameters of the PRS in cases and non-cases (putative non-affected controls). If the mean *m*_0_ and variance ${\sigma}_0^2$ of the PRS distribution in non-cases are unknown as unscreened population controls are used, they can be inferred from the means *m*_1_, *m*_*p*_ and variances ${\sigma}_1^2$, ${\sigma}_p^2$ of the PRS distributions in cases and in the population, respectively, and the disease prevalence *K* as

$$ {m}_0=\frac{m_p-K\ {m}_1}{1-K} $$

and

$$ {\sigma}_0^2=\frac{\sigma_p^2-K{\sigma}_1^2}{1-K}-\frac{K{\left({m}_p-{m}_1\right)}^2}{{\left(1-K\right)}^2} $$

(see Supplemental Note 1 for details).

### Estimation of the probability of disease development

By Bayesian inversion, a raw probability $\hat{P}$ to be affected by the disease can be inferred from an individual’s PRS value *x* and the distribution densities of PRS in cases, *p*_1_, and in controls, *p*_0_, as

2 $$ \hat{P}(x)=\frac{K\ {p}_1(x)}{K\ {p}_1(x)+\left(1-K\right){p}_0(x)} $$

However, $\hat{P}$ cannot be directly interpreted as a probability of disease. Logistic regression from case/control samples gives the probability of disease in the logistic model

3 $$ P(x)=\frac{1}{1+{e}^{-\left(\alpha +\beta x\right)}} $$

with coefficients *α*, *β* arising as regression parameters from the maximum likelihood estimate. We use linear regression with the logit link function, taking as data the log odds ratio corresponding to (2),

4 $$ y=\log\ \frac{K\ {p}_1(x)}{\left(1-K\right){p}_0(x)}, $$

at every PRS value *x* and the joint probability density of PRS in the population, *p*_*p*_(*x*) = *Kp*_1_(*x*) + (1 − *K*)*p*_0_(*x*), as weight. For normal densities *p*_1_ and *p*_0_, the coefficients *α*, *β* can be expressed as

5 $$ \alpha =\log \frac{K{\sigma}_0}{\left(1-K\right){\sigma}_1}+\frac{1}{2}\left(\left({r}_0-1\right)K+\left(1-{r}_1\right)\left(1-K\right)\right)-{m}_p\beta, \beta =\frac{m_1-{m}_0}{\sigma_p^2}\left(K\left(1-K\right)\left(\frac{r_0+{r}_1}{2}-1\right)+K\frac{\sigma_1^2}{\sigma_0^2}+\left(1-K\right)\frac{\sigma_0^2}{\sigma_1^2}\right) $$

where ${r}_1=\frac{\sigma_0^2+{\left({m}_1-{m}_0\right)}^2}{\sigma_1^2}$ , ${r}_0=\frac{\sigma_1^2+{\left({m}_1-{m}_0\right)}^2}{\sigma_0^2}$ , *m*_*p*_ = *Km*_1_ + (1 − *K*)*m*_0_, and${\sigma}_p^2=K\ {\sigma}_1^2+\left(1-K\right){\sigma}_0^2+K\left(1-K\right){\left({m}_1-{m}_0\right)}^2$ (see Supplemental Note 2 for details).

Formulae (5) determine the parameters of the logistic probability model (3) from the disease prevalence and the parameters of the distribution of PRS in cases and non-cases, dispensing with the need to obtain or simulate individual genotypes and perform logistic regression on the resulting PRS. They rely on the assumptions that the PRS distributions are normal and that the raw probability (2) represents well the fraction of cases for any value of PRS. For validation, we compared the outcome of (5) with the following three procedures of increasing abstraction, (a) simulation of genotypes in HWE with given MAF in cases and in non-cases and logistic regression of the resulting PRS, (b) sampling from normal distributions for PRS in cases and in non-cases with parameters ${m}_0,{m}_1,{\sigma}_0^2,{\sigma}_1^2$ and logistic regression, (c) sampling from the population distribution *p*_*p*_ and linear regression of the raw log odds ratio (4).

### Inclusion of rare variants in the probability

The effects of rare genetic variants with high (or medium) disease penetrance may be obscured if modelled as part of PRS including a large number of other SNPs, and the fraction of correctly identified cases carrying a rare mutation will be small in a sample and have little influence on the overall prediction accuracy. Therefore, it seems better to account for them at the level of the disease probability. Suppose we have the logistic regression model for the probability of disease *P*_*PRS*_ in terms of the PRS by formulae (3) and (5), excluding the rare variant from the calculation of the PRS. An individual with PRS value *x* who carries a rare genetic variant with intrinsic probability *p*_*rare*_ to cause the disease has, assuming the effects of the rare variant and of the polygenic risk are independent, the probability of disease

$$ P(x)={P}_{PRS}(x)+{p}_{rare}\left(1-{P}_{PRS}(x)\right)=\frac{1+{p}_{rare}\ {e}^{-\left(\alpha +\beta x\right)}}{1+{e}^{-\left(\alpha +\beta x\right)}} $$

where *x* is the PRS for the individual. For very rare variant alleles that do not affect the disease prevalence *K* in the population, the intrinsic probability can be estimated as

$$ {p}_{rare}=\frac{K\ \left( OR-1\right)}{K\ \left( OR-1\right)+1}, $$

where *OR* is the odds ratio (see Supplemental Note 3). The probability *P*(*x*) takes values between *p*_*rare*_ and 1, reflecting the liability of the rare variant to cause the disease even in absence of polygenic risk. In case of several rare variants with mutually independent effect and intrinsic probabilities *p*_*rare*, 1_, …, *p*_*rare*, *ν*_, the above formula can be applied with ${p}_{rare}=1-\prod \limits_{j=1}^{\nu}\left(1-{p}_{rare,j}\right)$. However, due to the assumption of very small allele frequencies, it is unlikely that an individual would carry more than one independent rare variant.

### Inclusion of APOE

It may be advantageous to treat a high-effect common variant such as *APOE* separately from the PRS. The distributions in cases and non-cases of a PRS formed from SNPs excluding *APOE* can be assumed to be approximately equal for carriers and non-carriers of the *APOE* risk allele. Considering formulae (5), the probability of disease as a function of PRS will then differ between the groups only due to the higher disease prevalence in carriers of the risk allele. Applying (5) with the disease prevalence for the different *APOE* genotypes, separate probability curves are obtained. The prevalence in different genotype groups is not usually directly available but can be inferred as follows from the overall prevalence *K*, the overall allele frequency *f* and the odds ratio *OR* for the variant, under the assumption of HWE both in the general population and in the subpopulation of non-cases. This assumption is justified when the disease prevalence in the population is low (e.g. 2% for AD), but problematic when it is high [24] (e.g. major depression 30%). The prevalence *K*_0_, *K*_1_ and *K*_2_ for carriers of non-risk homozygotes, heterozygotes and risk homozygotes, respectively, can be calculated as

$$ {K}_0=1-\frac{{\left(1-f-\nu \right)}^2}{\left(1-K\right){\left(1-f\right)}^2},{K}_1=1-\frac{\left(1-f-\nu \right)\left(\nu +f-K\right)}{\left(1-K\right)f\left(1-f\right)},{K}_2=1-\frac{{\left(\nu +f-K\right)}^2}{\left(1-K\right){f}^2}, $$

where

$\nu =\frac{b-\sqrt{b^2-16\left(1- OR\right)\left(1-f\right)K}}{4\ \left(1- OR\right)}$ with *b* = 2 (1 + (1 − *OR*)(*K* − *f*)) (see Supplemental Note 4).

### Standardisation of the probability curve

PRSs calculated from different sets of SNPs cannot be directly compared. We therefore standardise the PRS axis by expressing the PRS in terms of standard deviations difference from the population mean,

$$ {x}_{st}=\frac{x-{m}_p}{\sigma_p}, $$

where *x* is the PRS and *x*_*st*_ is the standardised PRS variable.

### Simulated and real data

Firstly, we simulated independent genotypes in a sample of 10,000 cases and 10,000 controls and used previously published effect sizes for genome-wide significant SNPs [2, 15]. We calculated an Oligogenic Risk Score (ORS) in the simulated sample using only 39 genome-wide significant SNPs (Supplementary Table 2, adopted from [15]), excluding the *APOE* proxy SNP (rs429358). The PRS was calculated for 10,039 SNPs, including the above 39 genome-wide significant SNPs and further 10,000 SNPs pruned for LD with *r*^2^ = 0.1 and allele frequencies and effect sizes taken from (2).

Secondly, to illustrate the probability of disease in the presence of rare variants, we used effect sizes for rare variants corresponding to the *APP, SORL1*, *TREM2*, *ABI3* and *PLCG2* genes [6, 7, 25]. We used the distribution parameters *m*_0_, *m*_1_ and ${\sigma}_0^2$,${\sigma}_1^2$ for ORS and PRS as reported in [23] and calculated the disease probabilities with the suggested formulae. To demonstrate the *APOE* modelling with our approach, we also took the distribution parameters of *APOE*, ORS and PRS from the real case/control study [23] (Supplemental Table 1).

The simulations and probability calculations were implemented with R-statistical software. The codes (Simulations.R and Probability.R) can be downloaded from https://github.com/DRI-Cardiff/AD-probability/.

## Results

As the validity of the formulae (5) was established by simulations (see Supplemental Figure 1), we used formulae (3) and (5) to calculate the probability of disease for an individual with PRS value *x*. This probability depends on the disease prevalence in the population of interest, e.g. the general population or a specific subpopulation. The prevalence of AD in the population depends strongly on age. Recent estimates show a 3%, 17% and 33% prevalence in the 65–74, 75–84 and 85+ age groups, respectively [26]. For illustration, we calculated the probability of AD for 2%, 10% and 30% prevalence during lifetime and in 65+ and 85+ age groups, respectively. The parameters of the PRS distributions were taken from a real case/control study [23].

**Fig. 1:**
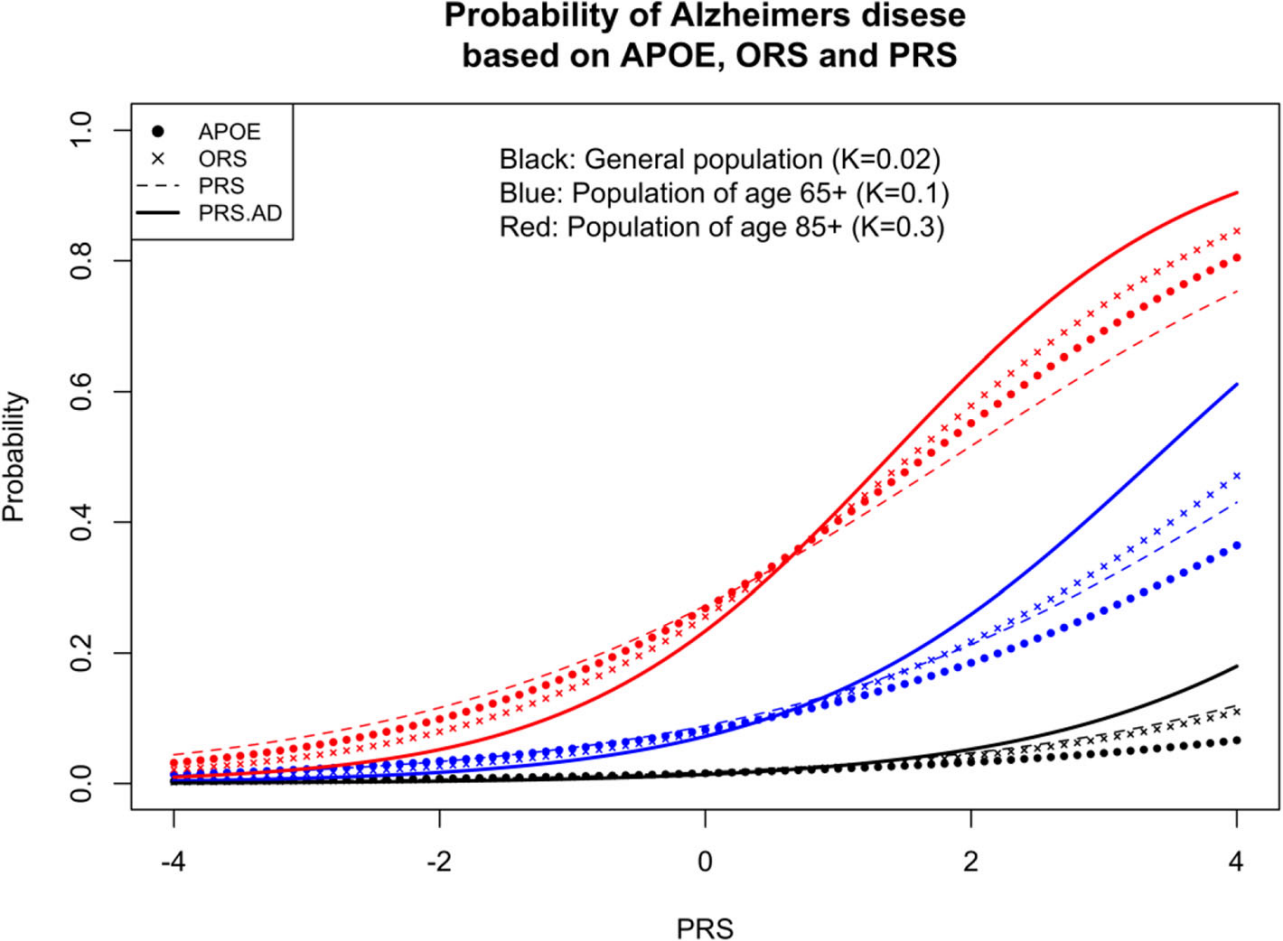
Probability of Alzheimer’s disease, ignoring change in APOE-*ε*4 allele frequency due to age. ORS - Oligogenic Risk Score including SNPs with a p-value threshold p_T_ ≤ 10^-5^. PRS - Polygenic Risk Score including SNPs with a p-value threshold p_T_ ≤ 0.1. PRS.AD - PRS calculated as a weighted sum of PRS.no.APOE, including SNPs with a p_T_ ≤ 0.1 excluding APOE region (CHR19:44.4-46.5), and APOE (ε2 + ε4), where APOE effects were weighted with effect sizes (B(ε2) = -0.47 and B(ε4) = 1.12) as in Kunkle et al. 2019 [2]

Figure 1 shows the dependency of the AD probability (*y*-axis) on standardised PRS (*x*-axis). The solid thick line corresponds to PRS.AD, calculated as weighted sum of *APOE* and PRS.noAPOE with the relative weight of *APOE* not taken directly from combining the corresponding effect sizes as a part of PRS but from bivariate logistic regression using *APOE* and PRS.noAPOE as predictors. The dashed line shows the probability of AD with PRS calculated in the standard way including all SNPs weighted with their single-SNP effect sizes. In the black and blue scenarios (corresponding to the lifetime and 65+ prevalences), the standard PRS shows clearer discrimination between low and high probabilities than *APOE* alone, somewhat similar to ORS. When the disease prevalence is high (red scenario), then PRS.AD is considerably more discriminative than PRS. This dramatic difference between PRS.AD and PRS is due to the fact that the means and variances for the latter are calculated in cases and controls, ignoring the change in *APOE*-*ε*4 allele frequency due to age, whereas PRS.AD indirectly accounts for it via the interplay of *APOE* and PRS.noAPOE.

**Fig. 2:**
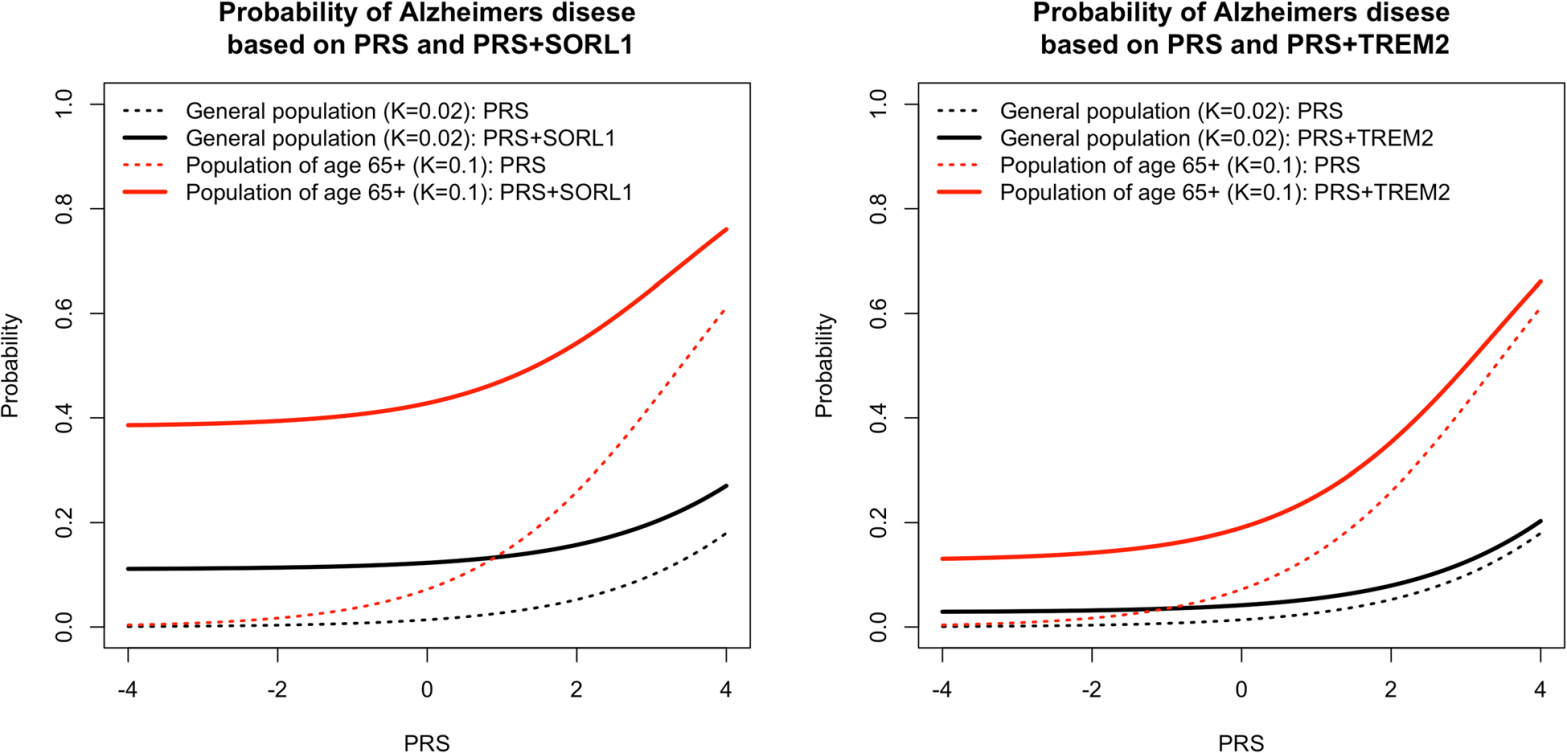
Combined probability of AD calculated with 2% lifetime prevalence of AD, 10% prevalence of AD in 65+ age group, including probability due to presence of a rare high-effect variant. The rare variant effect sizes and minor allele frequencies correspond to known variants in *SORL1* (OR = 7.2) [25] and *TREM2* (OR = 2.46) [7] genes. In age group 65+ (red) the presence of *SORL1* mutations (left) increases the AD probability from ~ 0 to 0.4 when PRS is the lowest and from 0.6 to 0.76 when the PRS is highest (solid line vs dashed line). For *TREM2* (right), these values are 0 to 0.13 (low PRS) and 0.6 to 0.66 (high PRS)

Figure 2 demonstrates the results of adding independent rare variant effects in *SORL1* and *TREM2* to the probability of the disease in both the general population (*K* = 0.02) and in the age group 65+ (*K* = 0.1). Both graphs show an elevated disease probability (solid lines), with lower values in the population (black) and higher values in the 65+ group. Dashed lines show the disease probability across the range of PRS if individuals have no rare risk variants. The results for rare variants such as in genes *APP*, *PSEN1* and *PSEN2* [27] are shown in Supplemental Figure 2.

**Fig. 3:**
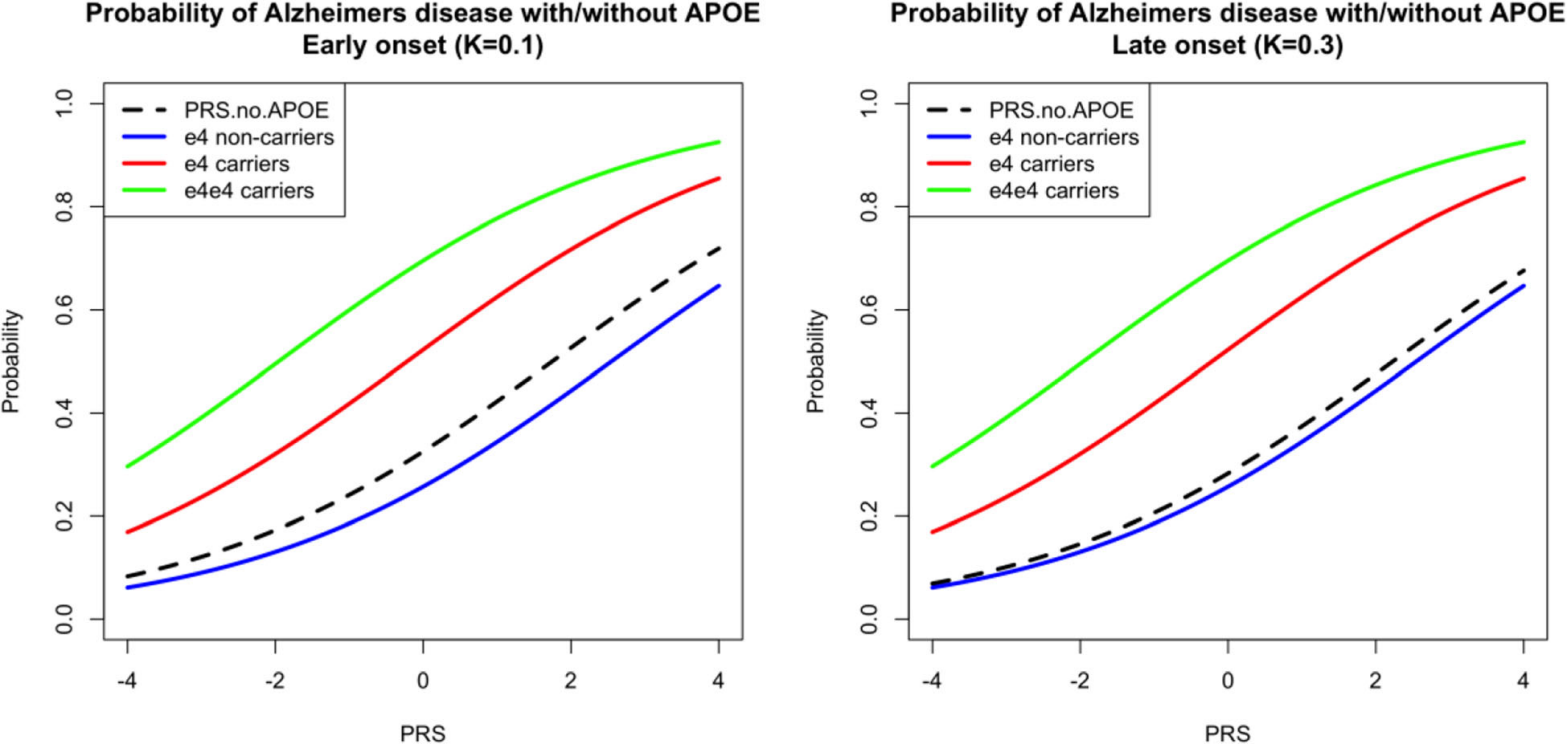
Modelling *APOE* separately, assuming the same effect of *APOE*-*ε*4 (OR ~ 3) in all age groups, and accounting for age related differences in frequency of *APOE*-*ε*4 allele (MAF = 0.18 in 55+ and MAF = 0.05 in 85+). In age group 65+ (left) the presence of *APOE*-ɛ4 allele increases the AD probability from 0.01 to 0.07 when PRS is the lowest and from 0.2 to 0.66 when the PRS is highest (top vs bottom lines). For 85+ age group (right), these values are 0 .06 to 0.3 (low PRS) and 0.65 to 0.92 (high PRS)

Finally, Figure 3 shows the probability of disease in early onset (left) and late onset (right) age groups. As expected, the late onset group shows elevated AD probability even if the PRS is low and *APOE ε*4 carriers show consistently higher probability than any other genotypes. The black dashed line shows the disease probability with PRS when the *APOE* region is excluded. It is slightly higher than for *APOE-*ε4 non-carriers (thin blue line) as excluding the *APOE* region removes the information whether the individuals have lower disease risk due to absence of *ε*4 or have protective *ε*2 alleles.

## Discussion

PRS do not directly indicate an individual’s liability to develop a disease, as they depend on a variety of study parameters such as the number and selection of SNPs included in their calculation and are therefore not comparable between different studies. For comparability, it is not sufficient to standardise the PRS against the population mean and variance; the difference in PRS means in cases and in non-cases is also essential for the interpretation of an individual’s PRS. Our proposed calculation of a probability of disease takes all of these properties of the PRS into account and provides a unified measure to assess a PRS value in view of the PRS distribution and disease prevalence in the population or subpopulation of interest. Of course, the choice of SNPs included in a PRS remains decisive for its indicative power.

While the probability curve as a function of standardised PRS can be calculated by logistic regression using genotyped case/control samples from the population of interest, we offer a method to achieve the same outcome using more easily available summary data. The theoretical formula (5) derives the parameters for the logistic probability function (3) from the disease prevalence and the PRS distribution parameters (mean and standard deviation) in cases and non-cases. This conveniently allows estimating an individual’s probability of disease from their PRS value using only a small set of parameters. We have shown that (5) gives a highly accurate proxy for case-control sampling of PRS and logistic regression if either the PRS aggregates a high number of SNPs or very highly associated SNPs (such as *APOE* variants for AD) are excluded from the PRS. If the PRS is calculated from a small number of SNPs including some that are highly associated, deviation from normality in the PRS may lead to some discrepancy, but the formula still gives a close approximation.

Moreover, the probability of disease framework allows for separate consideration of high-effect variants. It has been observed that inclusion of high-effect variants in the PRS calculation does not always give optimal results. The impact of common high-effect variants such as *APOE* for AD can vary due to confounders such as age stratification, so taking a summary effect size from a reference study may result in a suboptimal PRS; this becomes apparent when APOE and PRS are used as separate predictors in bivariate logistic regression. We propose including high-effect variants in two different ways in the calculation of disease probability.

The effect of rare, highly penetrant genetic variants tends to be masked by more common variants in the PRS. However, if they do not affect the disease prevalence in the population and act independently of the PRS, we can account for them directly as adding a certain intrinsic probability for carriers of the risk allele. The probability curve approaches this intrinsic probability for highly negative values of standardised PRS.

Common high-effect variants such as *APOE* for AD strongly affect the disease prevalence in the population and cannot be dealt with as above. Although *APOE* may not act independently from other genetic causal variants, a recent study [28] suggests that it is reasonable to assume that the distribution parameters for the PRS calculated without *APOE* are independent of the *APOE* genotype. We propose calculating separate probability curves for each *APOE* status, based on the disease prevalence calculated for each *APOE* status from the disease prevalence in the population, the risk allele frequency and the odds ratio. These data are available and can easily be further stratified into relevant subpopulations, e.g. by age for AD.

The disease probability allows comparison of PRS calculated from summary data of different reference studies and thus has the potential to be used in a clinical context to prioritise individuals for diagnostics and preventative intervention based on assessed risk of developing the disease.

There are other factors that can influence the disease development probability. For example, it has been reported that AD is more prevalent in women and PRS effects may also depend on gender [29]. Due to the lack of information on gender-interactions for genetic variants, incorporating gender information in the probability calculations may not be straightforward at present. However, in view of the emerging literature it is likely that this information will be available and reliable in future. It can then be incorporated in our calculations, e.g. by selecting the SNPs for the PRS in males and females separately and/or changing the disease prevalence not by age only, but by sex as well. It is possible to include other, non-genetic predictors. This can be achieved by adjusting the disease prevalence in different e.g. educational attainment groups.

Our approach can be used for other complex genetic disorders. For example, schizophrenia is a highly polygenic disorder [30] and has an increased burden of rare variants and CNVs [31]. It is, however, a neurodevelopmental disorder, and the disease prevalence does not depend on age. It also does not have strong genetic risk factors like *APOE* for AD. Therefore, the most relevant probability calculation approach for diseases like schizophrenia and depression is a combination of common and rare variants, while the method shown above for the inclusion of *APOE*-like variants is not required.

The proposed method relies on the availability of allele frequency and genetic effect size estimates derived from a representative reference population. Expanding this approach to other populations will be possible when the effect sizes of SNPs in other populations will be reliably identified and reported. They can then be used to estimate the disease probability in the relevant population. As in other complex genetic disorders, the disease risk estimates rely heavily on the individual SNP risk estimates and disease prevalence, which differ depending on the demographics, ethnicity and age groups.

## Limitation

A limitation of the present study is that it is based on sound, but theoretical principles, uses SNP and PRS characteristics from the literature and employs simulated data for validation. It remains to test and validate the theory directly in real datasets. Since we are dealing with rare variants, validation of this approach in real data with a sufficient level of confidence will require large population datasets like e.g. the UK BioBank [32]. However, the UK Biobank is not directly suitable to study neurodegenerative disorders, as the cohort is relatively young and only a small proportion of individuals manifest the disease. In addition, it does not provide phenotypic variables which are used to assess cognitive decline in dementia in clinical settings. Publicly available AD-specific datasets are typically small and not suitable to extract a reliable number of people carrying rare mutations (e.g. Alzheimer’s Disease Neuroimaging Initiative (ADNI) database (www.loni.ucla.edu/ADNI)). Nevertheless, the approach presented here can be used in small studies focusing on rare and common genetic variants, for example to identify individuals most at risk of developing the disease. It can easily and flexibly be updated as novel rare variants are discovered and as appropriate GWAS data become available for specific populations.

## Conclusions

The proposed method gives an estimate of the probability of developing AD based on an individual’s PRS, *APOE* genotype and the presence or absence of rare genetic variants associated with AD. The computational framework uses as reference data the means and standard deviations of the PRS employed in cases and in (either screened or population) controls and the disease prevalence. The disease prevalence varies considerably in different age and *APOE* genotype groups, and the present method allows for taking these differences into account in a natural and transparent way.

## Supplementary Information

**Additional file 1 **Supplemental Note 1 Estimate of distribution parameters with unscreened controls. Supplemental Note 2 Estimate of probability of disease by linear regression with logistic link function. Supplemental Note 3 Inclusion of rare variants in the probability. Supplemental Note 4 Inclusion of common variants of common with high effect. Supplemental Table 1 Mean and variance for the Alzheimer’s disease genetic risk scores. Supplementary Table 2 AD genome-wide significant SNPs (adopted from Andrewes et al 2020). Supplemental Figure 1 The comparison of the theoretical probability of disease using formulae (3), (5) with the probability of disease estimated by logistic regression of simulated genotypes. Supplemental Figure 2 Combined probability of AD calculated with 2% lifetime prevalence of AD, 10% prevalence of AD in 65+ age group, including probability due to presence of a rare variant. The effect size is set to OR=500 reflecting almost fully penetrant mutations in *APP*, *PSEN1*, and *PSEN2* genes.

## Data Availability

The relevant data and materials are available in the supplemental material.
