# Correction to: Probability of Alzheimer’s disease based on common and rare genetic variants

**DOI:** 10.1186/s13195-021-00898-1

**Published:** 2021-09-20

**Authors:** Valentina Escott-Price, Karl Michael Schmidt

**Affiliations:** 1 Dementia Research Institute, Division of Psychological Medicine and Clinical Neurosciences, School of Medicine, Cardiff University, Hadyn Ellis Building, Maindy Rd, Cardiff, CF24 4HQ UK; 2 School of Mathematics, Cardiff University, Senghennydd Road, Cardiff, CF24 4AG UK

**Correction to: Alz Res Therapy 13, 140 (2021)**

**https://doi.org/10.1186/s13195-021-00884-7**

Following the publication of the original article [1] the authors noticed that the published Fig. 3 is incorrect. The original article [1] has been updated.

Below is the correct Fig. 3.

**Fig. 3:**
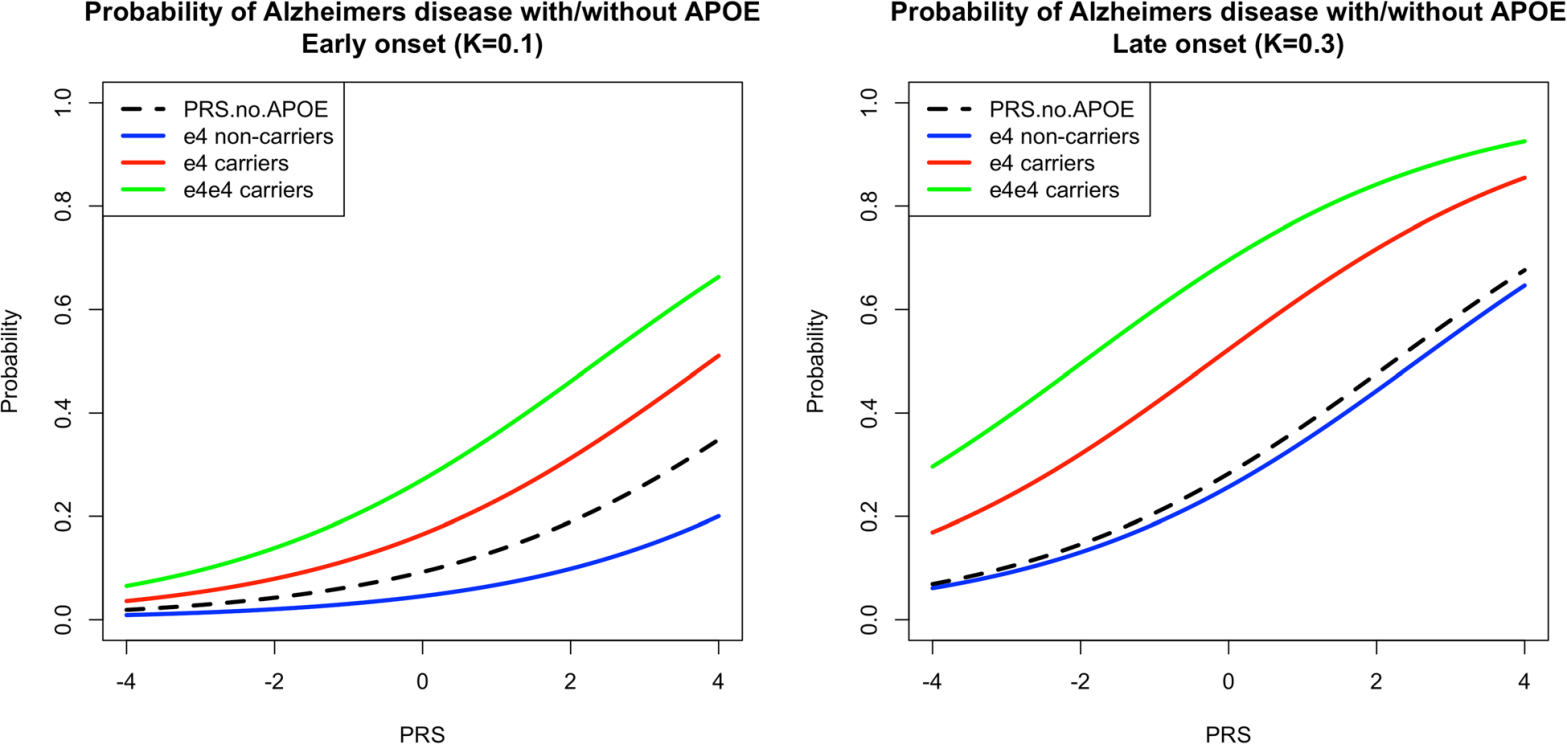
Modelling *APOE* separately, assuming the same effect of *APOE*-*ε*4 (OR ~ 3) in all age groups, and accounting for age related differences in frequency of *APOE*-*ε*4 allele (MAF = 0.18 in 55+ and MAF = 0.05 in 85+). In age group 65+ (left) the presence of *APOE*-ɛ4 allele increases the AD probability from 0.01 to 0.07 when PRS is the lowest and from 0.2 to 0.66 when the PRS is highest (top vs bottom lines). For 85+ age group (right), these values are 0.06 to 0.3 (low PRS) and 0.65 to 0.92 (high PRS)
