# Supplementary material for: Probability of Alzheimer’s disease based on common and rare genetic variants
Source: Alzheimers Res Ther. 2021 Aug 17;13:140. doi: 10.1186/s13195-021-00884-7 (PMC8369699; doi:10.1186/s13195-021-00884-7)
Supplement: Supplementary file 1 — Additional file 1 Supplemental Note 1 Estimate of distribution parameters with unscreened controls. Supplemental Note 2 Estimate of probability of disease by linear regression with logistic link function. Supplemental Note 3 Inclusion of rare variants in the probability. Supplemental Note 4 Inclusion of common variants of common with high effect. Supplemental Table 1 Mean and variance for the Alzheimer’s disease genetic risk scores. Supplementary Table 2 AD genome-wide significant SNPs (adopted from Andrewes et al 2020). Supplemental Figure 1 The comparison of the theoretical probability of disease using formulae (3), (5) with the probability of disease estimated by logistic regression of simulated genotypes. Supplemental Figure 2 Combined probability of AD calculated with 2% lifetime prevalence of AD, 10% prevalence of AD in 65+ age group, including probability due to presence of a rare variant. The effect size is set to OR=500 reflecting almost fully penetrant mutations in APP, PSEN1, and PSEN2 genes. [file 13195_2021_884_MOESM1_ESM.pdf]

RE: Probability of Alzheimer's disease based on common and rare genetic variants

Valentina Escott-Price and Karl Michael Schmidt for *Alzheimer's Research & Therapy*, 2021

Supplemental Note.

1. Estimate of distribution parameters with unscreened controls

Suppose we have a case sample and only a population (not a screened control) sample and from these estimated the means m_1, m_p and variances σ_1^2, σ_p^2 of the distribution of PRS in cases and in the population, respectively. Let p_1 be the density of the PRS distribution in cases, p_0 the density in non-cases and p_p the density in the population, with disease prevalence K. Then $p_p = K\,p_1 + (1-K)p_0$, which gives

$$p_0 = \frac{p_p - Kp_1}{1-K} \ .$$

Then we find the mean and variance of the distribution in non-cases as follows,

$$m_0 = \frac{m_p - Km_1}{1-K}$$

by linearity, and

$$\sigma_0^2 - m_0^2 = \frac{1}{1-K}\,(\sigma_p^2 - m_p^2) - \frac{K}{1-K}\,(\sigma_1^2 - m_1^2),$$

which upon using the above expression for m_0 gives

$$\sigma_0^2 = \frac{\sigma_p^2 - K\sigma_1^2}{1-K} - \frac{K\left(m_p - m_1\right)^2}{(1-K)^2} \ .$$

2. Estimate of probability of disease by linear regression with logistic link function

Suppose p_1 and p_0 are the probability densities of the PRS distribution in cases and in non-cases, respectively. The probability of disease can be estimated from these distributions and the disease prevalence K by logistic regression; in an interval of PRS between x and $x + dx$, we expect $N\,K\,p_1(x)\,dx$ cases and $N\,(1-K)p_0(x)\,dx$ non-cases in a (large) population of N individuals, and by integrating over x, can find the log-likelihood function for the logistic model of Eq. (3). As the maximum likelihood condition gives rise to implicit equations that cannot easily be solved, we use the alternative approach of linear regression with the logistic link function, taking as data the log odds ratio at each value x of the PRS and the probability density of the PRS in the population as weight. More precisely, we consider the linear regression model

$$y = \alpha + \beta\, x + \epsilon$$

with variables x, distributed according to the joint distribution of PRS in the population with density $p_p(x) = K\, p_1(x) + (1-K)p_0(x)$, and the logarithm of the odds ratio $y = \log \frac{K\, p_1(x)}{(1-K)p_0(x)}$, corresponding to the raw probability function

$$\hat{P}(x) = \frac{1}{1+e^{-y}} = \frac{Kp_1(x)}{Kp_1(x) + (1-K)p_0(x)}.$$

(We note that $\hat{P}$ is unsuitable for direct interpretation as a probability of disease, as it ignores the overall distribution of PRS in the population and generally does not increase with the PRS x throughout; indeed, for normal densities p_1 and p_0, the distribution with the larger variance will dominate both tails, independently of the difference of means.)

The least squares method gives the following estimates for the regression parameters,

$$\alpha = \bar{y} - \bar{x}\beta, \quad \beta = \frac{\overline{xy} - \bar{x}\,\bar{y}}{\overline{x^2} - \bar{x}^2},$$

where the bar denotes the sample average, here replaced with the population average. Here we have $\bar{x} = m_p = Km_1 + (1-K)m_p, \overline{x^2} - \bar{x}^2 = \sigma_p^2 = K\sigma_1^2 + (1-K)\sigma_0^2 + K(1-K)(m_1-m_0)^2$. Further, we obtain $\bar{y} = \log\frac{\mathrm{K}}{1-K} + \int_{-\infty}^{\infty} \log\frac{\mathrm{p_1(x)}}{p_0(x)}\, p_p(x)\, dx$ and $\overline{xy} = m_p \log\frac{\mathrm{K}}{1-K} + \int_{-\infty}^{\infty} x \log\frac{\mathrm{p_1(x)}}{p_0(x)}\, p_p(x)\, dx$. Hence

$$\beta = \frac{1}{\sigma_p^2} \int_{-\infty}^{\infty} \left(x - m_p\right)\, p_p(x) \log\frac{p_1(x)}{p_0(x)}\, dx,$$

$$\alpha = \log\frac{K}{1-K} + \int_{-\infty}^{\infty} p_p(x) \log\frac{p_1(x)}{p_0(x)}\, dx - m_p\beta.$$

The two integrals can be worked out explicitly for normal distributions

$$p_j(x) = \frac{1}{\sqrt{2\pi\sigma_j^2}}\, e^{-\frac{\left(x-m_j\right)^2}{2\sigma_j^2}} \quad (j \in (0,1)),$$

as for $j, k \in (0,1)$

$$\int_{-\infty}^{\infty} p_j(x) \log p_k(x)\, dx = \int_{-\infty}^{\infty} \frac{-\left(\sigma_j\xi + m_j - m_k\right)^2}{2\sigma_k^2}\, e^{\frac{-\xi^2}{2}} \frac{d\xi}{\sqrt{2\pi}} - \frac{1}{2}\log(2\pi\sigma_k^2)$$

$$= -\frac{1}{2}\left(\frac{\sigma_j^2 + \left(m_j - m_k\right)^2}{\sigma_k^2} + \log(2\pi\sigma_k^2)\right)$$

and

$$\int_{-\infty}^{\infty} (x - m\_p)\, p_j(x) \log p_k(x)\ dx$$

$$= \int_{-\infty}^{\infty} (\sigma_j \xi + m_j - m_p)\ \frac{-\left(\sigma_j \xi + m_j - m_k\right)^2}{2\sigma_k^2}\ e^{\frac{-\xi^2}{2}} \frac{d\xi}{\sqrt{2\pi}}$$

$$- \frac{m_j - m_p}{2} \log(2\pi\sigma_k^2)$$

$$= \left(m_k - m_j\right) \frac{\sigma_j^2}{\sigma_k^2} - \frac{m_j - m_p}{2} \left(\frac{\sigma_j^2 + \left(m_j - m_k\right)^2}{\sigma_k^2} + \log(2\pi\sigma_k^2) \right),$$

bearing in mind that the first and third moments of the standard normal distribution vanish and the second moment equals 1. Hence, abbreviating

$$r_1 = \frac{\sigma_0^2 + (m_0 - m_1)^2}{\sigma_1^2}\ , \quad r_0 = \frac{\sigma_1^2 + (m_0 - m_1)^2}{\sigma_0^2}\ ,$$

we obtain after some calculation

$$\beta = \frac{m_1 - m_0}{\sigma_p^2} \left(K(1 - K) \left(\frac{r_0 + r_1}{2} - 1 \right) + K \frac{\sigma_1^2}{\sigma_0^2} + (1 - K) \frac{\sigma_0^2}{\sigma_1^2} \right),$$

$$\alpha = \log \frac{K\ \sigma_0}{(1 - K)\sigma_1} + \frac{1}{2}\left((r_0 - 1)K + (1 - r_1)(1 - K)\right) - m_p \beta$$

as coefficients for the probability function (3). In the special case $\sigma_0^2 = \sigma_1^2$ the probability function from the regression model (3) with these values of α, β is equal to the raw probability function (2).

3. *Inclusion of rare variants in the probability*

Consider a rare variant SNP allele which appears to be causal by itself and thus contributes to the probability of developing the disease as a separate factor instead of as part of the polygenic risk. Assuming independence, the probability of disease P will then satisfy $1 - P = (1 - P_{PRS})(1 - p_{rare})$, so

$$P = P_{PRS} + p_{rare} - p_{rare} P_{PRS}\ ,$$

where P_{PRS} is the probability of disease due to the polygenic risk and p_{rare} is the intrinsic probability of the rare variant to cause the disorder.

Suppose the reference study had genotype counts n_0, n_1 and $n_2 = 0$ in cases, and m_0, m_1 and $m_2 = 0$ in controls. We assume that the variant is so rare that no individuals carry two risk alleles. Then the estimated probability of disease for non-carriers of the variant (in the sample) is $P_0 = \frac{n_0}{n_0 + m_0} = \frac{1}{1 + e^{-\alpha}}$ and the probability of disease for

carriers of the variant is $P_1 = \frac{n_1}{n_1+m_1} = \frac{1}{1+e^{-\alpha-\beta}}$; here β is the log odds ratio. Using the above formula for the combined probability and $P_1 = \frac{1}{1+\frac{1-P_0}{P_0}e^{-\beta}}$, we find

$$p_{rare} = \frac{P_1 - P_0}{1 - P_0} = \frac{P_0\left(e^{\beta} - 1\right)}{P_0(e^{\beta} - 1) + 1}$$

for the intrinsic probability. In order to estimate the intrinsic probability for the rare variant to cause the disease in the population, we use the disease prevalence K as a proxy for the background probability P_0, assuming the variant to be so rare that it does not affect the population prevalence, and as $e^{\beta} = OR$, we obtain the estimate

$$p_{rare} = \frac{K(OR - 1)}{K(OR - 1) + 1}$$

and hence the combined probability of disease

$$P = P_{PRS} + \frac{K(OR - 1)}{K(OR - 1) + 1}\ (1 - P_{PRS})\ .$$

If there are several rare variants with intrinsic probabilities $p_{rare,1}, \dots, p_{rare,v}$, then assuming their effects are mutually independent, the joint probability for disease caused by the rare variants will be $p_{rare} = 1 - \prod_{j=1}^{v}(1 - p_{rare,j})$. However, due to the assumption of very small allele frequencies, it is unlikely that an individual would carry more than one independent rare variant.

4. *Inclusion of common variants of common with high effect*

We propose calculating the probability of disease for a high-effect common variant such as *APOE* for AD separately per genotype, based on the respective disease prevalence, instead of including the variant in the PRS. The disease prevalence in carriers and non-carriers of the risk allele are not usually known but can be calculated from the genotype counts in cases and non-cases. If the raw genotype data are not available, these counts can be inferred from general information as follows.

Suppose there is a population of N individuals, the risk allele has frequency f in the population and odds ratio OR, and the disease prevalence is K. Then the allele counts $\widetilde{n_0}, \widetilde{n_1}$ in cases and $\widetilde{m_0}, \widetilde{m_1}$ in cases and in controls, respectively, satisfy the equations $\widetilde{n_0} + \widetilde{m_0} = 2(1-f)N, \widetilde{n_1} + \widetilde{m_1} = 2fN, \widetilde{n_0} + \widetilde{n_1} = 2KN, \widetilde{m_0} + \widetilde{m_1} = 2(1-K)N$ and $OR = \frac{\widetilde{n_1}\,\widetilde{m_0}}{\widetilde{m_1}\,\widetilde{n_0}}$. We now express $\widetilde{n_0}$ by the first equation, substitute for $\widetilde{m_0}$ from the

odds ratio, replace $\widetilde{m_1}$ by the second equation and then express the remaining unknown $\widetilde{n_1}$ by the third equation. This gives a quadratic equation for $\widetilde{n_0}$,

$$0 = (1 - OR)\,\widetilde{n_0}^2 - 2N\big(1 + (1 - OR)(K - f)\big)\,\widetilde{n_0} + 4K(1 - f)N^2$$

and hence

$$\widetilde{n_0} = \frac{b - \sqrt{b^2 - 16(1 - OR)(1 - f)K}}{2\,(1 - OR)} N$$

with $b = 2(1 + (1 - OR)(K - f))$. This, by means of the above set of equations, determines all allele counts uniquely. In particular, For the genotype counts, we assume HWE in the population to obtain the equations $n_0 = \frac{\widetilde{n_0} - n_1}{2}$, $n_2 = \frac{\widetilde{n_1} - n_1}{2}$, $m_1 = 2f(1 - f)N - n_1$, $m_0 = (1 - f)^2 N - n_0$, $m_2 = f^2\, N - n_2$. Here n_1 is still a free parameter; in fact, the given data do not determine the genotype counts uniquely. If we further assume that HWE also holds in non-cases, an assumption which is not unrealistic if the prevalence is not too high, we have the additional equation $4m_0 m_2 = m_1^2$, and substitution from the last three equations above yields

$$n_1 = 2f(1 - f)N - \frac{\widetilde{m_0}\,\widetilde{m_1}}{\widetilde{m_0} + \widetilde{m_1}} = 2f(1 - f)N - \frac{2(1 - f - \nu)(f - K + \nu)}{1 - K} N,$$

where $\nu = \frac{\widetilde{n_0}}{2N}$, so the prevalence for genotype 1 is

$$K_1 = \frac{n_1}{n_1 + m_1} = \frac{n_1}{2f(1 - f)N} = 1 - \frac{(1 - f - \nu)(f - K + \nu)}{(1 - K)f(1 - f)}.$$

Further, the prevalence for genotypes 0 and 2, respectively, is calculated as

$$K_0 = \frac{n_0}{n_0 + m_0} = \frac{\widetilde{n_0} - n_1}{2(1 - f)^2 N} = 1 - \frac{(\nu - 1 + f)^2}{(1 - K)(1 - f)^2}$$

and

$$K_2 = \frac{n_2}{n_2 + m_2} = \frac{\widetilde{n_1} - n_1}{2f^2 N} = 1 - \frac{(\nu + f - K)^2}{(1 - K)f^2}.$$

Supplemental figure 1. The comparison of the theoretical probability of disease using formulae (3), (5) with the probability of disease estimated by logistic regression of simulated genotypes.

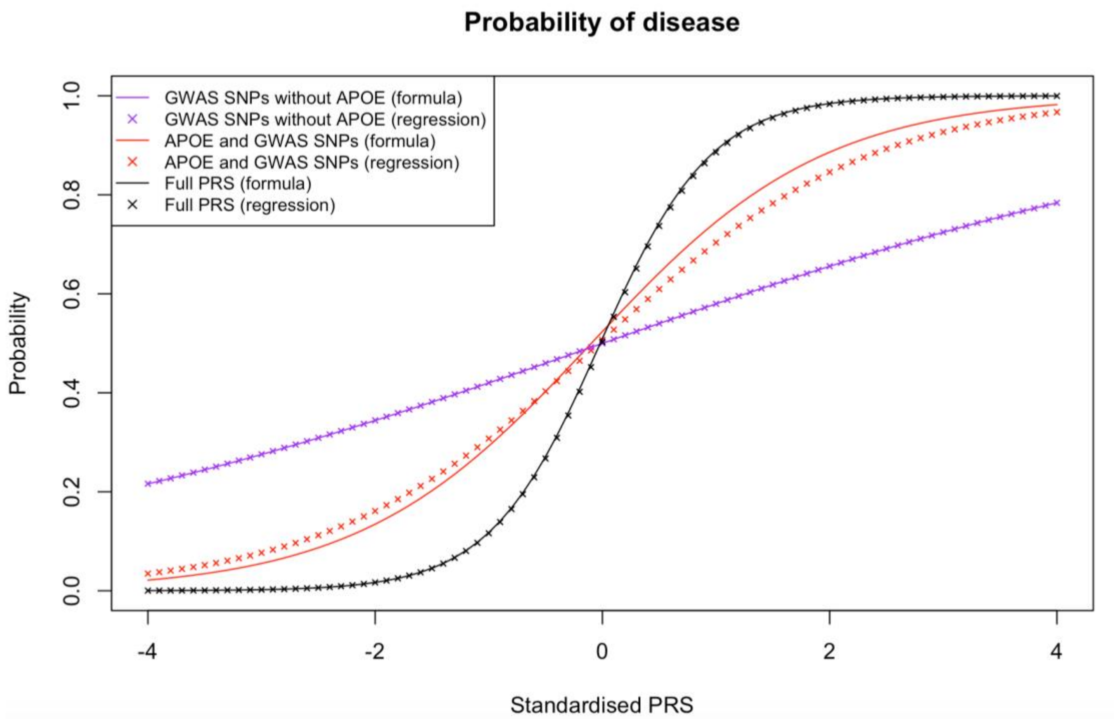

Legend: For the logistic regression results, the data were simulated for the *APOE* proxy SNP and for the ORS constructed from the 39 GWAS significant SNPs. The independent SNPs were simulated for 10,000 cases and 10,000 controls and added to the PRS weighted by their log odds ratios as effect sizes (see Methods). The regression curves show a fair fit with the theoretical curve in the cases where the PRS is formed from *APOE* and the 39 GWAS significant SNPs (red curve and points); the other approximations (logistic regression from normally distributed samples for PRS in cases and in non-cases, and linear regression of the raw probability function from a normally distributed sample for PRS in the population) lie close to the shown theoretical curve. In the simulations, the distributions of PRS in cases and in non-cases are skewed away from normality when *APOE* is included with a moderate number of less highly associated SNPs (histograms not shown); this plausibly explains the discrepancy. When *APOE* is omitted from the PRS or when it is included with a large number of SNPs of small effect size (in this figure N SNPs=100,000), the fit is perfect (purple and black curves and points).

Supplemental Figure 2. Combined probability of AD calculated with 2% lifetime prevalence of AD, 10% prevalence of AD in 65+ age group, including probability due to presence of a rare variant. The effect size is set to OR=500 reflecting almost fully penetrant mutations in *APP*, *PSEN1*, and *PSEN2* [1] genes.

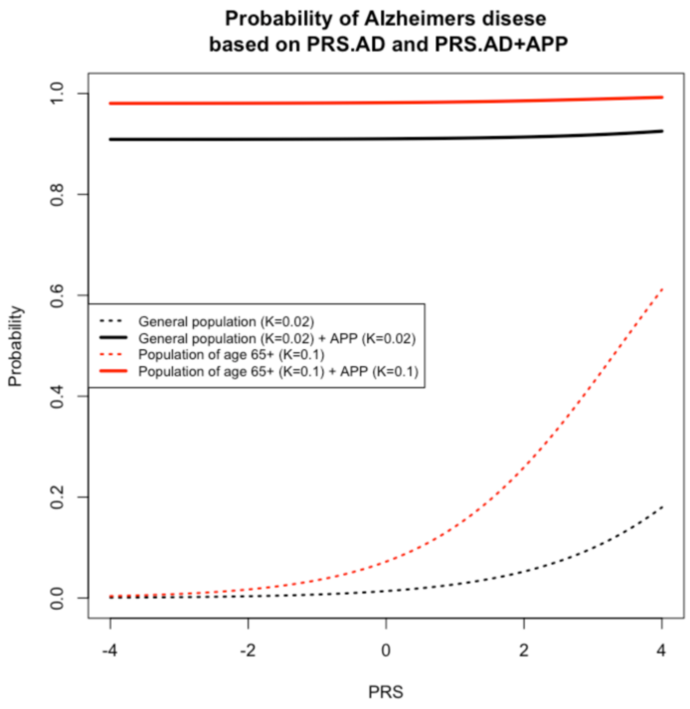

Caption: Since these mutations are associated with very early onset (55 years and below, sometimes as low as 25 years old) and explain only 5–10% of the occurrence of early-onset AD, this figure uses the prevalence K=0.02, the lifetime prevalence of AD.

Supplemental Table 1. Mean and variance for the Alzheimer's disease genetic risk scores adapted from [2].

	Cases		Controls	
	m_1	σ_1^2	m_0	σ_0^2
ORS	0.478	1.54	-0.335	0.87
PRS	0.385	1.19	-0.193	1.08
PRS.no.APOE	0.320	1.19	-0.136	1.14
PRS.AD	0.630	1.44	-0.370	0.89

ORS (Oligogenic Risk Score): genetic risk score including SNPs with p-value threshold $p \leq 1x10^{-5}$.

PRS (Polygenic Risk Score): genetic risk score including SNPs with p-value threshold $p \leq 0.1$.

PRS.no.APOE: Polygenic Risk Score including SNPs with a p value threshold $p \leq 0.1$ and excluding SNPs in the *APOE* region (chr19:44.4-46.5Mb).

PRS.AD: PRS calculated as a weighted sum of PRS.no.APOE and *APOE*(ε2+ε4), where *APOE* effects were weighted with effect sizes (B(ε2)=-0.47 and B(ε4)=1.12) as in Kunkle et al. 2019 [3].

Supplemental Table 2. AD genome-wide significant SNPs (adopted from Andrews et al 2020[4]).

rsID	Chr	Nearest Gene	AltAlleleFreq	OR	p-value
rs4575098	1	ADAMTS4	0.239	1.02	2.1e–10
rs4844610	1	CR1	0.78	0.85	3.6e–24
rs6733839	2	BIN1	0.395	1.2	2.4e–69
rs35349669	2	INPP5D	0.512	1.07	3.6e–11
rs184384746	3	HESX1	0.002	1.21	1.2e–08
rs6448453	4	CLNK	0.772	0.99	1.9e–09
rs190982	5	MEF2C	0.663	1.08	3.2e–08
rs34855541	6	HLA-DRB1	0.135	0.9	9.5e–15
rs187370608	6	UNC5CL	0.001	1.25	1.5e–16
rs9381563	6	CD2AP	0.656	0.93	5.8e–14
rs2718058	7	GPR141	0.347	0.93	4.8e–09
rs1476679	7	ZCWPW1	0.73	1.1	9.9e–19
rs10808026	7	EPHA1	0.204	0.91	1.1e–14
rs114360492	7	CNTNAP2	0.001	1.19	2.1e–09
rs4236673	8	CLU	0.611	1.12	1.1e–28
rs7920721	10	ECHDC3	0.393	1.08	1.8e–11
rs3740688	11	SPI1	0.526	1.09	5.5e–13
rs7933202	11	MS4A6A	0.341	0.89	1.9e–19
rs10792832	11	PICALM	0.667	1.13	5.1e–36
rs11218343	11	SORL1	0.034	0.81	4.6e–17
rs17125924	14	FERMT2	0.099	1.12	1.3e–11
rs12590654	14	SLC24A4	0.347	0.92	8.2e–12
rs59685680	15	SPPL2A	0.247	0.93	9.2e–09
rs593742	15	ADAM10	0.298	0.93	2.8e–11
rs117618017	15	APH1B	0.107	1.02	3.30e-08
rs59735493	16	KAT8	0.324	0.99	4.0e–08
rs7185636	16	IQCK	0.156	0.92	2.40e-08
rs4985556	16	IL34	0.088	1.09	3.7e–08
rs62039712	16	WWOX	0.094	1.16	3.7e–08
rs12444183	16	PLCG2	0.657	1.06	3.2e–08
rs7225151	17	SCIMP	0.118	1.1	6.1e–12
rs28394864	17	RP11-81K2.1	0.471	1.01	1.9e–08
rs2526380	17	BZRAP1	0.449	0.97	2.6e–08
rs138190086	17	CYB561	0.017	1.25	1.9e–09
rs76726049	18	ALPK2	0.011	1.06	3.3e–08
rs3752246	19	ABCA7	0.838	0.87	3.1e–16
**rs429358***	**19**	**APOE**	**0.216**	**3.32**	**1.2e–881**
rs12459419	19	CD33	0.336	0.99	6.3e–09
rs6069736	20	CSTF1	0.088	0.89	2.0e–10
rs2830500	21	ADAMTS1	0.336	0.93	2.6e–08

* - APOE SNP effect size is taken from Kunkle et al 2019[3].

Supplemental references

1. Van Cauwenberghe C, Van Broeckhoven C, Sleegers K. The genetic landscape of Alzheimer disease: clinical implications and perspectives. *Genet Med.* 2016;18(5):421-430.
2. Leonenko G, Baker E, Stevenson-Hoare J, et al. Identifying individuals with high risk of Alzheimer’s disease using polygenic risk scores. *Nature Communications.* 2021;(under review).
3. Kunkle BW, Grenier-Boley B, Sims R, et al. Genetic meta-analysis of diagnosed Alzheimer's disease identifies new risk loci and implicates Abeta, tau, immunity and lipid processing. *Nat Genet.* 2019;51(3):414-430.
4. Andrews SJ, Fulton-Howard B, Goate A. Interpretation of risk loci from genome-wide association studies of Alzheimer's disease. *Lancet Neurol.* 2020;19(4):326-335.
